# Circular RNAs in Hematopoiesis with a Focus on Acute Myeloid Leukemia and Myelodysplastic Syndrome

**DOI:** 10.3390/ijms21175972

**Published:** 2020-08-19

**Authors:** Michaela Dostalova Merkerova, Zdenek Krejcik, Katarina Szikszai, David Kundrat

**Affiliations:** Institute of Hematology and Blood Transfusion, 128 20 Prague, Czech Republic; zdenek.krejcik@uhkt.cz (Z.K.); katarina.szikszai@uhkt.cz (K.S.); david.kundrat@uhkt.cz (D.K.)

**Keywords:** circular RNAs, hematopoiesis, acute myeloid leukemia, myelodysplastic syndrome

## Abstract

Circular RNAs (circRNAs) constitute a recently recognized group of noncoding transcripts that function as posttranscriptional regulators of gene expression at a new level. Recent developments in experimental methods together with rapidly evolving bioinformatics approaches have accelerated the exploration of circRNAs. The differentiation of hematopoietic stem cells into a broad spectrum of specialized blood lineages is a tightly regulated process that depends on a multitude of factors, including circRNAs. However, despite the growing number of circRNAs described to date, the roles of the majority of them in hematopoiesis remain unknown. Given their stability and disease-specific expression, circRNAs have been acknowledged as novel promising biomarkers and therapeutic targets. In this paper, the biogenesis, characteristics, and roles of circRNAs are reviewed with an emphasis on their currently recognized or presumed involvement in hematopoiesis, especially in acute myeloid leukemia and myelodysplastic syndrome.

## 1. Introduction

In the last decade, the development of high-throughput RNA-seq technologies accelerated the discovery of various noncoding RNAs (ncRNAs). Although they do not encode proteins, ncRNAs account for 98% of all human RNAs [1] and seem to be functional in numerous cellular processes. NcRNAs can be categorized into several classes, such as ribosomal RNAs (rRNAs), transfer RNAs (tRNAs), microRNAs (miRNAs), long noncoding RNAs (lncRNAs), PIWI-interacting RNAs (piRNAs), small nuclear RNAs (snRNAs), small nucleolar RNAs (snoRNAs), and circular RNAs (circRNAs).

CircRNAs are non-polyadenylated single-stranded closed-loop RNA structures. Naturally occurring circRNAs were first described several decades ago when uncoated covalently closed RNA molecules were discovered in plants and termed viroids [2]. They were originally considered mere byproducts of transcription. In the 90s, a number of publications reported the circularization of processed gene transcripts [3,4,5,6]. For example, development-specific processing of the SRY gene was demonstrated. Although the protein product of SRY is a key molecule in the early development of testes, an abundance of an unusual circular SRY transcript (cSRY) was observed in adult testes [7]. This circRNA was observed in cytoplasm but was not definitively bound to polysomes, limiting its translational potential [7], and it seemed to function by sponging miR-138 [8].

Commonly, circRNAs are deconvoluted and identified by next-generation sequencing (NGS), with over 10,000 human circRNAs annotated thus far [9]. They were recognized as significant RNA species, and their high functional relevance was demonstrated [8]. However, it remains unclear whether the majority of circRNAs represent splicing byproducts without function or are produced in a regulated manner to carry out specific cellular functions.

Myelodysplastic syndrome (MDS) represents a spectrum of hematopoietic stem cell (HSC) disorders characterized by ineffective hematopoiesis, peripheral blood cytopenia, and a tendency for transformation into acute myeloid leukemia (AML). Therefore, MDS is often considered a preleukemic disorder. MDS is a dynamic disease in which clonal evolution triggers disease onset and progression. Clonal evolution is assumed to be a multistep process in which several successive genetic abnormalities are acquired by normal HSCs, leading to the expansion of an MDS clone and subsequent transformation to AML. Despite considerable advances in the understanding of the roles of various classes of noncoding RNAs in MDS, the roles of circRNAs in the onset or progression of this disease remain unclear.

In this review, we describe specific features and various roles of this novel class of molecules and put them into the context of hematopoiesis, with particular emphasis on myelodysplasia.

## 2. CircRNA Features and Biogenesis

Although generally expressed at low levels, global expression profiling has revealed that circRNAs comprise numerous classes of transcripts with diverse expression patterns among cell types [10] and are particularly enriched in the nervous system [11]. The expression level of some circRNAs can be higher than that of their linear cognates [12,13]. The abundance of circRNAs is probably reinforced by their extreme stability, as their circular and non-polyadenylated structure renders circRNAs naturally resistant to exonucleases. The circRNA half-life was estimated to exceed 24–48 h [14]. Interestingly, circRNAs exhibit a degree of evolutionary conservation at the nucleotide level across eukaryotes [12]. This conservation reinforces the likelihood of a relevant and significant role for RNA circularization.

CircRNAs originate from transcripts that are joined head-to-tail via a process called backsplicing. The covalent circularization of RNA molecules results in a novel backward fusion of two gene segments [15]. The backsplicing process thus ligates a downstream splice donor site, in reverse, with an upstream splice acceptor site such that the circRNA strands are in the opposite direction of those in canonically spliced linear RNAs. Canonical pre-mRNA splicing is catalyzed by the spliceosomal machinery, which removes introns and joins exons, and it has been shown that both canonical splice signals and canonical spliceosomal machinery are required for backsplice circularization [16,17]. The fact that the canonical spliceosome is involved in backsplicing suggests that circRNA formation competes with the formation of linear cognates [16].

**Figure 1:**
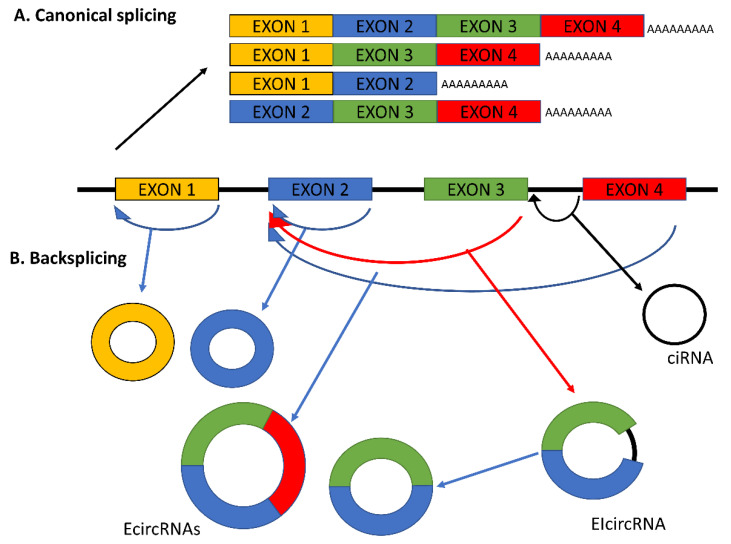
Schema of pre-mRNA splicing. (**A**) Canonical splicing with alternative splice products. (**B**) Backsplicing with the major types of circular RNAs (circRNAs) generated by the junctions of exons, introns, or both. (1) Exonic circRNAs (EcircRNAs). (2) Exon-intron circRNAs (EIcircRNAs) that are circularized with introns retained between exons. (3) Circularized intron RNAs (ciRNAs). Several circRNA isoforms can be produced from a single gene locus.

CircRNAs can be formed by the circularization of a one or more exons (exonic circRNAs or ecircRNAs) [18], both exons and introns (exon-intron circRNAs or EIcircRNAs) [19], or intron(s) only (circularized intron RNAs or ciRNAs) [20] (Figure 1). In addition to these major forms of circRNAs, several other types of circularized transcripts have been identified, such as intergenic circRNAs [21] and fusion circRNAs (f-circRNAs), which are generated from translocated or rearranged chromosomes [22]. The majority of circRNAs belong to the ecircRNA class [23]. The sizes of circRNAs vary, ranging from a few hundred to thousands of nucleotides. With respect to the formation process, several circRNA isoforms can be produced from a single gene locus, a type of alternative circularization, and these isoforms can differ by expression profile under different conditions and/or function [24].

The biogenesis of circRNAs is influenced by different factors in *cis* and *trans.* Regarding *cis* regulation, it has been shown that parental genes of circRNAs tend to have more active promoters. For example, significantly higher levels of the histone acetylation mark H3K27Ac and lower levels of DNA methylation were observed in promoter regions of genes from which circRNAs are formed compared to genes from which linear RNAs, but no detectable circRNAs, are generated [14]. The backsplicing process is facilitated by repetitive sequences in flanking introns that are in reverse-complement orientation. These elements enable backsplicing through the formation of hairpin structures that bring the circularizing exons close to each other [25,26].

In addition to *cis* regulatory factors, multiple RNA-binding proteins, such as splicing factors, play important roles in backsplicing in *trans*. Several studies have reported that different proteins bind to pre-mRNAs as a bridge between flanking introns, thus facilitating the process of circularization. In humans, many of the repetitive structures overlap with Alu elements [12,27]. In this context, it has been shown that backsplicing can be regulated by DHX9 and ADAR proteins to control circRNA formation [27,28]. DHX9 is a nuclear RNA helicase that binds specifically to inverted repeat Alu elements. The loss of DHX9 leads to an increase in the number of circRNA-producing genes and the amount of circRNAs formed [28]. ADAR1 is an RNA-binding protein that functions in RNA editing. Intron bracketing circRNAs are highly enriched in RNA editing or hyperediting, and the knockdown of ADAR1 significantly and specifically upregulated circRNA expression [27]. Interestingly, codepletion of ADAR and DHX9 augmented double-stranded RNA accumulation defects, leading to increased circRNA production and thus revealing a functional link between these two enzymes [28].

Another protein that plays a role in circRNA biogenesis is MBNL1. MBNL1 binds to its own pre-mRNA to bridge two flanking introns, bringing them close together to induce backsplicing and resulting in upregulated circRNA formation from its own RNA [16]. Further, splicing factor ESRP1 can promote circularization through intronic binding sites flanking the *circular form of BIRC6* in human embryonic stem cells [29]. Splicing factors have been shown not only to promote circRNA biogenesis but also to repress circRNA formation. For example, the disruption of the serine-arginine splicing factors SRSF1/6/11 or hnRNP Hrb27C enhances the expression of the circular form of laccase2 in *Drosophila* [30]. However, despite some advances in understanding the nature of circRNAs, the number of molecules involved in the process of RNA circularization and the associated regulatory networks controlling circRNA function remain largely unclear.

## 3. Molecular Functions of circRNAs

Several functions have been attributed to circRNAs. Emerging evidence indicates that circRNAs constitute one of multiple layers of posttranscriptional regulation of gene expression. They can serve as miRNA sponges [8,21]; interact with RNA-binding proteins (RBPs) to sequester them from their target RNAs or mediate their subcellular localization [31]; modify the stability of mRNAs by forming duplex structures [32]; modulate protein–protein interactions by acting as dynamic scaffolds [33]. They can even become translated into proteins through cap-independent initiation of translation [34,35].

The most recognized mechanism of action of circRNAs is their function as a miRNA sponge. Through the formation of a circRNA-miRNA duplex, the miRNA that is complementary to a circRNA is sequestered, which allows the binding of translational machinery to the mRNA targeted by this miRNA, leading to de-repression of a silenced gene. The first circRNA observed acting as a miRNA sponge was CDR1as, which has more than 70 conserved binding sites for miRNA-7 and depletes this miRNA in neuronal tissues [36]. Since this initial discovery, many circRNA sponges of miRNA have been described. Although miRNA sponging is the classical function of circRNAs, the universality of this model is being questioned because a recent study showed that most circRNAs cannot function as miRNA sponges [37].

Although circRNAs are generally considered noncoding molecules, recent studies have indicated that some cytoplasmic circRNAs can be effectively translated into detectable peptides [34,35]. CircRNAs may contain an open reading frame, but they lack the essential components necessary for translation, such as a poly(A) tail and a 7-methylguanosine cap. Due to the lack of a 5′ end, their translation can be initiated only through a cap-independent mechanism that requires an internal ribosomal entry site (IRES). Indeed, it has recently been reported that some circRNAs can be translated in vivo via different IRESs [35]. However, IRESs are infrequent in eukaryotic transcriptomes, which casts doubts on the scope of circRNA translation.

## 4. Methods and Databases for circRNA Exploration

Currently, the number of newly identified circRNAs is rapidly growing; however, the functions of the majority of these species remain unknown. The recent development of experimental methods for the exploration of circRNAs together with rapidly evolving bioinformatics approaches deepen our understanding of circRNA biogenesis and functionality. In this section, we summarize the methods available to detect and study circRNAs.

As a convenient starting point to obtain current knowledge on circRNAs, several databases and web resources are available to explore known circRNAs, predict their binding partners, and identify putative functions in different tissues or diseases (Table 1). The two most commonly used databases are probably circBase [9], which provides circRNA sequences and data on tissue specificity based on publicly available RNA-seq data, and CircInteractome [38], which also offers a tool to design divergent primers for PCR detection of individual circRNAs. Although the nomenclature of circRNAs has not yet been standardized across different databases, circBase assigns circRNAs unique circRNA IDs, which have also been used in the CircInteractome database.

The NGS of circRNAs (circ-Seq) enables the identification and expression profiling of thousands of circRNA molecules. In contrast to mRNAs, circRNAs do not contain a poly-A tail; therefore, they are not detected by the most widely used RNA-seq methods, which are based on poly-A enrichment. Although circRNAs are retained in rRNA-depleted RNA samples, in-depth sequencing, e.g., 100–150 million reads, must be performed with these NGS libraries [44]. As an alternative to rRNA depletion, the circRNA fraction can be highly enriched by treatment with 3′→5′ exoribonuclease RNase R, which eliminates most linear RNAs but enables circRNAs to remain intact [10]. However, some (usually highly structured) RNAs may remain refractory to RNase R digestion [45]; in contrast, many circRNAs are sensitive to RNase R under some conditions [12,46]. Therefore, various new approaches for circRNA enrichment are rapidly evolving. For example, a method termed RPAD is based on initial RNase R treatment followed by polyadenylation and poly(A)^+^ RNA depletion. These joint methods drastically deplete linear RNAs and lead to the isolation of highly pure circRNAs [47]. There are also electrophoresis-based methods for circRNA enrichment, namely, an agarose gel assay trap and a two-dimensional PAGE method [46,48]. Alternatively, known circRNAs (and their corresponding linear cognates) can be detected by designing a targeted circRNA AmpliSeq panel [49]. In these cases, circRNAs must be fragmented or nicked before library preparation to open the circle and allow adapter ligation and random priming.

In RNA-seq data, circRNAs can be identified by their unique backsplice junctions, which result in the alignment of chimeric reads, a feature that distinguishes them from linear RNAs. Multiple computational tools have recently emerged for the detection of these backsplice junctions within sequencing data. Most of these tools were developed as open-source software by researchers and therefore differ in many regards. A list of the most relevant computational tools is presented in Table 2. The tools can be separated into several categories based on the method used for circRNA detection. The “candidate-based” approach depends on predicted circRNA sequences, which are constructed based on the provided circRNA annotation information before the reads are aligned. This method is utilized by the KNIFE [50] and NCLscan [51] tools, for example, that were previously described in [46]. The “fragment based” strategy [52] is based on reads that were mapped to the genome for identifying backsplice junctions based on multiple-split reads in the mapping information. This approach is used in algorithms such as CIRCexplorer [53], MapSplice [54], DCC [55], and circtools [56]. Two of the algorithms listed in Table 2 are based on unique strategies. CIRI2 exploits paired chiastic clipping (PCC) signals found in mapping information of locally aligned reads to find possible circRNA candidates [57]. CircMarker focuses solely on circRNA detection and does not use read mapping; in contrast, it analyzes short sequence segments, called k-mers, avoiding the need to reconstruct entire circRNA sequences. Identified k-mers are then compared to the table of k-mers obtained from the transcriptome annotation files provided [58]. Since the detection methods of these algorithms vary considerably, differences in the number of circRNAs detected can be expected, and the possibility for false discoveries must be considered. A number of previous papers have focused on the comparison of these algorithms [59,60], and they each concluded that no single method is preeminent. Moreover, these studies emphasized that circRNA annotation should be handled with care and that several algorithms should ideally be combined to achieve reliable predictions.

In addition to NGS, the microarray approach is another high-throughput expression profiling method used to detect levels of tens of thousands of established circRNAs. As it is based on probes specifically designed to recognize junction sequences, this method does not distinguish between different circRNAs that share a junction. As in circRNA-seq, usually an RNase R-treated circRNA-enriched fraction is hybridized onto an array [61].

Another probe-based method is Northern blot analysis, where either short junction-specific or longer probes complementary to most of a circRNA sequence can be used [62]. Under specific conditions, this analysis can even be applied to determine circRNA size, isoform, processing, sequence, and abundance [35].

RNA fluorescence in situ hybridization (RNA-FISH) probes targeting circRNA junctions can also be used to detect and quantify the abundance and localization of circRNAs [46,62]; however, as only one or a few FISH probes within the junction sequence can be used for visualization, circRNA signals are usually weaker than those of linear RNAs for which multiple probes can be applied.

To validate the high-throughput methods, reverse transcription followed by quantitative polymerase chain reaction (RT-qPCR) analysis is usually applied [63,64]. For the qPCR step, divergent primers (designed, for example, by CircInteractome [38]) spanning the circRNA junction are essential. As divergent primers do not amplify linear RNAs, RNase R treatment is not always necessary, although it is often used. Additionally, PCR products can be subsequently Sanger sequenced to further verify the junction sequences and obtain the full sequences of given circRNAs [63,65].

To measure circRNAs in low abundance or to quantify circRNA copies in absolute numbers, the digital droplet PCR (ddPCR) method can be advantageous. Based on this technology, template nucleic acids (cDNA) are partitioned into tens of thousands of nanoliter-sized droplets for subsequent PCR amplification, and the final ratio of positive to negative droplets is analyzed to determine circRNA concentration [66]. This method has been successfully used in several circRNA studies [67,68].

## 5. Methods for Studying circRNA Functions

To better recognize the mechanisms of circRNA effects, it is essential to study the molecular networks of circRNAs with their interacting partners, such as miRNAs and RBPs. For these purposes, circRNA affinity pulldown assays are performed using biotinylated antisense oligomers specifically designed against the junction sequence of a target circRNA. Using streptavidin-coated beads, the biotinylated probes are pulled down together with the target circRNA and its interacting molecules. Then, the pulldown proteins can be analyzed by mass spectrometry and Western blotting, and the RNA components can be studied by RT-qPCR or sequencing [69]. Another approach for studying circRNA interactions with specific proteins is ribonucleoprotein immunoprecipitation (RIP). An antibody against an RBP of interest is used for immunoprecipitation, and RBP-associated RNAs, including circRNAs, are then detected by RNA-seq, Northern blot, or RT-qPCR [69].

The major challenge to study circRNA function is knowing the way to affect their levels specifically and effectively. The first successful steps for mimicking circRNAs have been taken. For example, circRNAs synthesized in vitro were introduced into eukaryotic cells to study the activation of the innate immune response induced by the circRNAs [70]. Further, exogenous circRNAs were also transfected into eukaryotic cells to induce the stable expression of a functional protein encoded by the exogenous circRNAs [71]. An interesting work was reported by Meng et al. [72], who designed artificial small circular single-stranded DNA (CSSD) containing multiple binding sites for miR-9. Transfection of this CSSD resulted in increased expression of the KLF17, CDH1, and LASS2 tumor suppressors because of sponged miR-9, which in turn led to the inhibition of tumor proliferation and metastasis and promoted apoptosis. Using this approach, this group showed that a single CSSD can simultaneously promote the inhibition of multiple tumor suppressor genes. In addition, the CSSD was more stable and effective than currently used miRNA inhibitors, and transfecting CSSDs via nanoparticles substantially improved the efficiency of their delivery [72].

On the other hand, depleting circRNAs in a specific way without affecting existing genes and their linear transcripts is challenging. To achieve substantial circRNA-specific knockdown via RNA interference or CRISPR/Cas9 technology, siRNA molecules or CRISPR/Cas9 guide RNA sequences must be designed to the backsplice junction site, which is unique to a given circRNA. For example, Piwecka et al. successfully removed circular CDR1as by CRISPR/Cas9 from the mouse genome, and then, they deregulated the expression of miR-7 and thus affected brain functions [73].

## 6. CircRNAs in Hematopoiesis

HSC differentiation into a broad spectrum of specialized blood cells is a tightly regulated process that depends on a multitude of transcription factors and other molecules, including noncoding RNAs. As circRNAs can drive a subset of cellular functions, their expression in different blood cell populations is being evaluated to determine their involvement in hematopoiesis. However, most roles of circRNAs in the regulation of HSC differentiation remain to be revealed.

The first study on a circRNA in the context of hematopoiesis was published in 1998 by Candas et al. [74], who reported that splicing of the MLL gene is an extremely complex process that often results in scrambled transcripts and circRNAs in both normal and leukemic cells. With the emergence of the NGS era, research on the blood transcriptome has been rapidly accelerated, and the catalog of hematopoiesis-related circRNAs has begun to grow rapidly. In 2012, Salzman et al. [13] performed deep RNA sequencing in hyperdiploid B-lineage acute lymphoblastic leukemia cells and observed a noncanonical mode of RNA splicing that resulted in the formation of various circRNA isoforms. They demonstrated that this process occurs frequently and is not a specific feature of leukemia cells but is a general feature of the gene expression program in human cells.

An RNA-seq analysis showed that whole blood is very rich in circRNAs, even comparable to the circRNA abundance in the cerebellum [75]. However, whole blood is composed of a complex mixture of different cell types, which are presumed to contain different circRNA contents. Thus, a more detailed description of circRNA profiles of specific hematopoietic cell lineages is needed. For example, a comparison of CD34+, CD19+, neutrophils and HEK293 (human embryonic kidney cells) was reported by Memczak et al. [21]. The study detected a total of 1950 circRNAs, of which 939 were exclusively expressed in the CD19+ cells, 333 in the CD34+, 194 in the neutrophils and 60 in the HEK293 cells, and only 19 circRNAs were common to the different cell populations. The results of the study clearly demonstrated that circRNAs are cell-type-specific and are expressed in a developmental stage-related manner. Further, Nicolet et al. [76] provided a more comprehensive analysis of circRNA expression during hematopoiesis. They showed that, similar to differentiating neurons [11], the expression levels of circRNAs increase overall during hematopoietic differentiation. However, some circRNAs were found to be preferentially expressed in hematopoietic progenitors, while others were enriched in a specific differentiated lineage. For example, the expression level of circ-FIRRE was high in all progenitors except for the common lymphoid progenitors; circ-BACH1 (exons 2–4) was preferentially expressed in HSCs and multipotent progenitors; circ-FNDC3B in NK cells; circ-MYBL1 and circ-SLFN12L in T cells and NK cells; circ-AKT3 and circ-CCDC91 in lymphoid cells; and circ-BACH1 (exons 3–4) in monocytes. The highest numbers of circRNAs were found in fully differentiated enucleated cells, platelets, and erythrocytes [64]. Whether the high prevalence of circRNAs in enucleated cells is merely a result of their high stability or whether circRNAs can be generated outside the nucleus is uncertain. Nevertheless, it is tempting to speculate that these enucleated blood cells can use circRNAs as templates for translation to maintain their functions and respond to environmental factors. Moreover, specific circRNAs may be released from these cells to transmit signals to other cells via microvesicles [77].

Due to their high stability [14], circRNAs have considerable potential to be used as cancer biomarkers. Their abnormal tissue- and time-specific expression [10] may play crucial roles in disease development and progression. Several recent studies revealed a close association of particular circRNAs with hematopoietic malignancies, especially AML [78,79,80,81,82,83,84,85,86]. As AML is the malignancy most related to MDS, we summarize the roles of particular circRNAs in this disease in more detail.

## 7. CircRNAs in AML

One of the first circRNAs associated with AML was the circular form of NPM1 mRNA; hsa_circ_0075001. It is highly expressed in the leukemia cells of AML patients and shows no or minimal blast maturation (M0 or M1 subtypes according to the FAB classification), whereas its expression is significantly lower in the subtypes with a more mature blast population (M2, M4 and M5) [78]. Somatic mutations in the NPM1 gene are the most common recurring genetic changes in AML. The expression of hsa_circ_0075001 positively correlates with total NPM1 expression but is independent of NPM1 mutational status. Interestingly, high expression of hsa_circ_0075001 is closely associated with lower expression of genes involved in the Toll-like receptor (TLR) signaling pathway. As TLR activity is associated with leukemic stem cell survival and differentiation, hsa_circ_0075001 may be a potential biomarker for the classification and risk stratification of AML [78].

Another circRNA highly expressed in AML is circ-DLEU2. Wu et al. [79] reported that circ-DLEU2 induces the expression of PRKACB by sponging miRNA-496, consequently promoting the proliferation of leukemia cells and inhibiting apoptosis in AML tumors.

Circ-ANAPC7 is an additional promising biomarker for AML diagnosis. Chen et al. [80] showed that this circRNA might participate in AML pathogenesis by acting as a sponge for the miR-181 family. By molecular interaction modeling and gene set enrichment analysis, they revealed that circ-ANAPC7 is associated with cancer-related pathways and that most of its target genes are involved in biological processes closely associated with AML tumorigenesis [80].

Another circRNA related to AML cells is hsa_circ_100290 (circularized transcript of the SLC30A7 gene). This circRNA is downregulated not only in AML [81] but also in other cancers, suppressing the proliferation, invasion, and migration of cancer cells [82]. Fan et al. [81] found that hsa_circ_100290 expression is increased in AML and that its silencing induces cell cycle arrest and apoptosis. They described hsa_circ_100290 function as a sponge of miR-203 and noted that it is coexpressed with RAB10 (a target of miR-203) [81].

In de novo patients with AML, Wu et al. [83] observed significantly upregulated levels of a circular form of vimentin mRNA (circVIM). Its overexpression seems to predict poor clinical outcomes and is closely associated with shorter overall survival of AML patients [83].

A similar impact on the outcome of AML patients was reported for hsa_circ_0009910, a circRNA formed from a transcript of the MFN2 gene [84]. Patients with high expression of this circRNA had a lower overall survival rate than those with low expression. Moreover, its knockout inhibited the proliferation and initiated the apoptosis of AML cells by sponging miR-20a-5p, highlighting the diagnostic and therapeutic potential of this circRNA in AML [84].

On the other hand, hsa_circ_0004277 (circularized transcript of the WDR37 gene) is downregulated in newly diagnosed AML patients [85]. Patients who achieve complete remission after treatment reestablish a normal expression level, showing no difference with the control group. However, in relapsed or refractory patients, the downregulation of hsa_circ_0004277 is resumed. Interestingly, the expression of hsa_circ_0004277 and WDR37 was found to be positively correlated during different AML stages [85].

Shang et al. [86] focused on the roles of circRNAs in chemoresistance in AML. They identified significant overexpression of circPAN3 in patients with refractory and recurrent AML compared to the level expressed by chemosensitive patients. Subsequent mechanistic experiments showed that the downregulation of circPAN3 decreased the expression of XIAP, but this effect was counteracted by specific inhibitors of miR-153–3p and miR-183–5p. The authors concluded that circPAN3 can serve as a novel marker predicting the clinical efficacy of chemotherapy in AML patients and can serve as a potential target for reversing drug resistance in AML [86].

**Figure 2:**
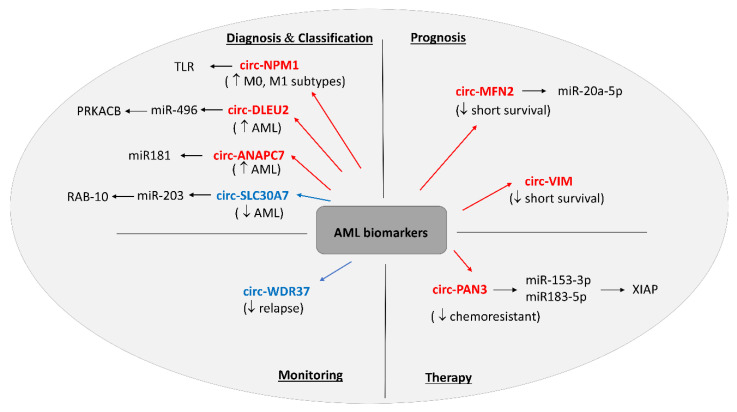
Summary of circRNAs suggested as potential acute myeloid leukemia (AML) biomarkers based on [78,79,80,81,82,83,84]. The deregulations of circRNAs are marked in red (upregulation) and blue (downregulation). The diagram also illustrates the circRNA–miRNA–mRNA axes.

To summarize, intense research on circRNAs in AML has led to our rapidly evolving knowledge of the importance of RNA circularization in hematological malignancies in general. It clearly shows that circRNA expression is closely associated with the onset of leukemia, its progression, and resistance to treatment, and undoubtedly, these data demonstrate the high potential of circRNAs in the prediction and monitoring of the course of the disease (Figure 2).

## 8. CircRNAs and Splicing in MDS

Significant progress in the study of MDS pathogenesis has been realized in recent years. A number of breakthrough studies have described the presence of multiple somatic mutations in MDS [87,88]. These mutations occur in key components of the spliceosome machinery, regulators of DNA methylation, chromatin modification, transcription, signal transduction, and cell cycle control. Mutations in RNA splicing factors constitute the most common class of genetic alterations in MDS, as they are present in 50–60% of MDS patients. They are highly specific for myelodysplasia and closely related disorders such as chronic myelomonocytic leukemia (CMML). The SF3B1 gene is mutated in approximately 15–28% of all MDS patients and is thus the most frequently mutated gene described in this disease. In particular, SF3B1 mutations are present in more than 70% of patients with MDS with ring sideroblasts (MDS-RS). Other mutations are found in the splicing-related genes SRSF2 (10–15%), U2AF1 (5–16%), and ZRSR2 (3–11%) and, to a lesser extent, in several other splicing-related factors. Regarding the outcome, patients with SF3B1 mutations showed significantly longer survival and a lower probability of disease progression. In contrast, mutations in the SRSF2 gene are likely to predict shorter survival times and a higher probability of disease progression [89].

All of these frequently mutated splicing factors represent essential components of the spliceosome; for example, the SF3B1 protein is a core component of the U2 small nuclear ribonucleoprotein (snRNP) that is involved in the recognition of the branch point and the 3′ splice site and prevents premature nucleophilic attack at the site by the splicing machinery [89].

These mutations frequently occur early in the pathogenesis of MDS and are also enriched in clonal hematopoiesis of indeterminate potential, suggesting their role in disease initiation [90]. Splicing factor mutations are heterozygous and largely mutually exclusive to each other. The feature of mutual exclusivity suggests that a cell with a splicing factor mutation cannot tolerate further perturbation of splicing induced by a second mutation in this pathway. In contrast, the cooccurrence of splicing mutations with mutations in epigenetic modifiers (DNMT3A or ASXL1) has been frequently described [89]. It is noteworthy that, in addition to the abovementioned splicing factors, mutations in several other genes, such as the epigenetic modifiers DNMT3A [91] and TET2 [92], have been described as related to aberrant splicing in MDS.

Although RNA splicing has been recognized as one of the key processes for MDS development, the impact of mutated splicing factors on circRNA deregulation has not been studied thus far. Given the role of mRNA splicing machinery in the formation of circRNAs, an attractive hypothesis suggests that these mutant proteins alter the amounts of circRNAs and their linear RNA cognates, thereby resulting in a myelodysplastic phenotype. Although this hypothesis has not yet been validated, a link between circRNA expression and SF3B1 depletion was reported by Liang et al. [93], who found that depletion or pharmacological inhibition of the spliceosome components of the SF3B or SF3A complexes caused an increase in circRNA levels coupled to a reduction in linear mRNA levels. The steady-state output of the reporter gene thus was increasingly predominated by circRNAs when early steps in spliceosome assembly were slowed or inhibited [93].

Nevertheless, the degree of deregulation of circRNA expression in MDS remains a mere presumption based on its similarity in other hematological diseases, especially AML, and the exact impact of splicing mutations on circRNA expression is still unknown.

## 9. Current Challenges and Future Prospects of circRNA Research

The enormous progress made in the field of circRNA research highlighted a significant potential for the future applicability of circularized nucleic acids in medicine and life science technologies. Due to their high stability and specificity, circRNAs have rapidly become promising biomarkers for the development and progression of various diseases. The expression dynamics of circRNAs is different from that of currently used disease biomarkers (i.e., protein coding genes or other noncoding RNA species). Thus, combination of various classes of molecular markers might increase the accuracy and specificity of present diagnostic and prognostic models. However, these expectations have raised some important challenges that need to be comprehensively considered before specific features of circRNAs confer benefits.

In the context of technical challenges, the experimental methodology needs to be fine-tuned. As the expression of a circRNA is closely connected to the expression of its host gene, it is rather difficult to define the functional aspects of circRNAs using standard techniques. Further, we urgently need a unified nomenclature system for circRNAs to be able to incorporate information on circRNAs into RNA databases, such as RefSeq and the UCSC genome browser. Most likely, the naming of circRNAs in future research should be based on the host gene along with the term “circ”. However, the explicit determination of circRNA isoforms (i.e., specification of the precise site of the backsplice junction and involvement of different exons/introns) needs to be in some form of their numbering, e.g., circNPM1–1 would denote the first isoform of a circular transcript of the NMP1 gene. Finally, more gold standard studies for data comparison are increasingly necessary to reliably understand circRNA biology.

In addition to the utilization of circRNAs as helpful disease biomarkers, it is expected that they will be exploited for therapeutic purposes. One of the best understood features of circRNAs is their ability to sponge miRNAs. Numerous tumor suppressive/oncogenic miRNAs have been reported to contribute to the development and progression of leukemia. Thus, the possibility of interfering with the processes associated with leukemia via the circRNA–miRNA–mRNA axis seems very promising. A substantial benefit of the circRNA-based approach is that one circRNA generally contains multiple miRNA-binding sites (MREs); thus, affecting the expression of a circRNA may offer treatment advantages over a single miRNA/gene. Depending on the type of sponged miRNAs, circRNAs can function as oncogenes or tumor-suppressors. Depleting an oncogenic circRNA via RNA interference or CRISPR/Cas9 technology can promote the protective effect of the corresponding miRNA in suppressing oncogenes. In contrast, transfection of an artificially synthetized circRNA into leukemia cells may sponge oncogenic miRNAs, leading to impaired leukemogenesis. These potential options may lead to the development of novel therapeutic strategies through circRNA-based miRNA sponging mechanisms that prevent tumor development.

## 10. Conclusions

Descriptions of circRNA molecules forced us to reconsider a simplistic definition of the term “gene”. This reevaluation enabled us to support the concept that genes are complex transcriptional units rather than simple pieces of a DNA sequence encoding a protein. It is now apparent that one locus can be transcribed under different conditions in considerably different ways, performing distinct biological roles.

Our current understanding of HSC differentiation and the abnormalities leading to leukemogenesis mainly originated from knowledge of transcription factors. However, recent discoveries of somatic mutations in RNA splicing genes in MDS have revealed that abnormal functions related to RNA splicing may be involved in leukemogenesis. These discoveries were quite unexpected, as this type of mutation is generally uncommon in cancer. Although studies seeking to understand the effects of these mutations on RNA splicing at a global- and transcript-specific level in MDS are currently ongoing, circRNAs have largely fallen outside this line of scientific inquiry thus far. To finally understand the roles of aberrant splicing in myelodysplasia, further studies should therefore focus on questions such as, how do RNA splicing mutations affect the expression of circRNAs, and how do circRNAs contribute to the pathogenesis of MDS?

## Figures and Tables

**Table 1:** CircRNA databases.

Database	Content	Webpage	Reference
CircBase	CircRNA sequences, descriptions, and genomic positions for six species, sequence-based search, public circRNA data sets.	http://www.circbase.org	[9]
CIRCpedia v2	CircRNA annotations from over 180 RNA-seq data sets, computational tools for the comparison of circRNA expression.	http://www.picb.ac.cn/rnomics/circpedia	[39]
CircInteractome	Prediction and mapping of binding sites for RNA-binding proteins (RBPs) and miRNAs on reported circRNAs, a tool to design divergent primers for PCR detection of individual circRNAs.	http://circinteractome.nia.nih.gov	[38]
CirclncRNAnet	Functional networks of *circRNAs/lncRNAs* from user-defined gene expression data.	http://app.cgu.edu.tw/circlnc	[40]
CircR2Disease	Experimentally supported associations between circRNAs and diseases.	http://bioinfo.snnu.edu.cn/CircR2Disease	[41]
TSCD	Global view of tissue-specific circRNAs in main tissues of human and mouse.	http://gb.whu.edu.cn/TSCD	[42]
CSCD	Information on cancer-specific circRNAs.	http://gb.whu.edu.cn/CSCD	[43]

**Table 2:** Selected computational tools for circRNA detection in RNA-seq data.

Software	Short Description	Aligner Used	Detection Method	Reference
CIRI2	Extracts paired chiastic clipping (PCC) signals from locally aligned reads.	BWA-MEM	PCC and local alignment	[57]
CIRCexplorer	Python-based tool; detects backsplicing junctions using TopHat-Fusion algorithm.	TopHat	Fragment based	[53]
CircMarker	Comparison of short sequence segments (k-mers).	-	k-mer comparison	[58]
circtools	Based on DCC analysis; offers a wide variety of visualization tools.	STAR	Fragment based	[56]
DCC	Utilizes STAR alignments and applies several logical filters during detection process.	STAR	Fragment based	[55]
KNIFE	Uses reads based on previous filtering to find viable hits in circRNA databases and offers de novo detection of circRNAs.	Bowtie/Bowtie2	Candidate based	[50]
MapSplice	Identifies multiple types of splice junctions; de novo splice mapping software.	Bowtie	Fragment based	[54]
NCLscan	Uses several stepwise alignments to eliminate false positives; detects all noncollinear transcripts.	BWA, Blat, NovoAlign	Candidate based	[51]
